# Fungal arthritis with adjacent osteomyelitis caused by *Candida pelliculosa*: a case report

**DOI:** 10.1186/s12879-020-05171-8

**Published:** 2020-06-22

**Authors:** Kwang Yun Song, Chulmin Park, Ji-Hyun Byun, Hye-Sun Chun, Jung-Hyun Choi, Eun Hee Han, Seung Ok Lee, Yeonjeong Jeong, Youn Jeong Kim, Si-Hyun Kim

**Affiliations:** 1 Department of Orthopedic Surgery, College of Medicine, The Catholic University of Korea, Seoul, Republic of Korea; 2 Vaccine Bio Research Institute, College of Medicine, The Catholic University of Korea, Seoul, Republic of Korea; 3 Department of Internal Medicine, College of Medicine, The Catholic University of Korea, Seoul, Republic of Korea; 4 Department of Laboratory Medicine, College of Medicine, The Catholic University of Korea, Seoul, Republic of Korea; 5 Division of Infectious Diseases, Department of Internal Medicine, Incheon St. Mary’s Hospital, College of Medicine, The Catholic University of Korea, 56 Dongsu-ro, Bupyeong-gu, Incheon, 403-720 Republic of Korea

**Keywords:** Arthritis, Osteomyelitis, *Candida*, Micafungin

## Abstract

**Background:**

*Candida* sp. osteoarticular infection is rare and most often due to hematogenous seeding during an episode of candidemia in immunocompromised patients. However, the diagnosis can be delayed in patients with subtle symptoms and signs of joint infection without a concurrent episode of candidemia.

**Case presentation:**

A 75-year-old woman presented with a three-year history of pain and swelling of the left knee. *Candida pelliculosa* was detected from the intraoperative tissue when the patient had undergone left total knee arthroplasty 32 months ago, but no antifungal treatment was performed. One year after the total knee arthroplasty, *C. pelliculosa* was repeatedly isolated from the left knee synovial fluid and antifungal treatment comprising amphotericin B deoxycholate and fluconazole was administered. However, joint infection had extended to the adjacent bone and led to progressive joint destruction. The patient underwent surgery for prosthesis removal and received prolonged antifungal treatment with micafungin and fluconazole.

**Conclusions:**

This case shows that *C. pelliculosa*, an extremely rare non-*Candida albicans sp.,* can cause fungal arthritis and lead to irreversible joint destruction owing to delayed diagnosis and treatment.

## Background

Infectious arthritis may be caused by various pathogens including bacteria, viruses, fungi, and parasites. Although bacterial arthritis is the most common joint infection, *Candida*, *Cryptococcus*, and *Aspergillus* species and other molds are capable of causing fungal arthritis, particularly in immunocompromised or critically ill patients [1, 2]. Fungal arthritis is an uncommon but challenging clinical condition, and *Candida* spp. are the most common cause.

*Candida pelliculosa* is a rare human pathogen. It is a ubiquitous yeast usually found in fruits, grains, soil, plants, and warm-blooded animals. The first case of invasive infection by this pathogen in humans was reported in 1953, and since then there have been a few cases of fungemia, endophthalmitis, and meningitis caused by *C. pelliculosa* [3–6]. Thus far, cases of osteoarticular infection caused by *C. pelliculosa* are extremely rare [7]. Herein, we report a case of fungal arthritis with adjacent osteomyelitis caused by *C. pelliculosa* in an elderly patient with no underlying immunosuppressive condition.

## Case presentation

A 75-year-old woman presented with a 3-year history of pain and swelling of the left knee, with the pain particularly intensifying over the past month. Because oral medications and intra-articular injections of unidentified agents did not help improve her symptoms, the patient had undergone left total knee arthroplasty at a local hospital 32 months ago. *C. pelliculosa* was isolated from the tissue collected by intraoperative biopsy, but no antifungal treatment was initiated. One year after the total knee arthroplasty, *C. pelliculosa* was repeatedly isolated from the left knee synovial fluid, and the patient received the following antifungal agents for 9 weeks: intravenous amphotericin B deoxycholate (0.5 mg/kg/day) for 4 weeks, then intravenous fluconazole (6 mg/kg/day) for 3 weeks, followed by oral fluconazole (4 mg/kg/day) for 2 weeks. However, progressive bone loss around the prosthetic components was seen on follow-up radiographs, and the patient was referred to a tertiary hospital. The patient had no other significant medical history.

**Fig. 1:**
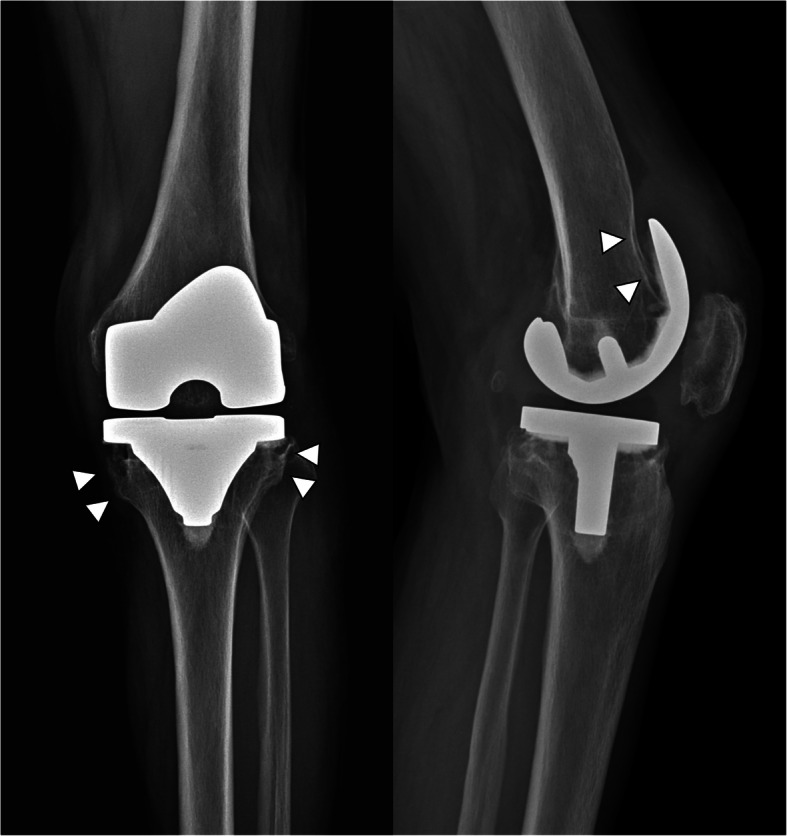
Plain radiography image of anteroposterior and lateral view of the left knee at admission, showing bony resorption of proximal tibia and distal femur (arrow heads)

**Fig. 2:**
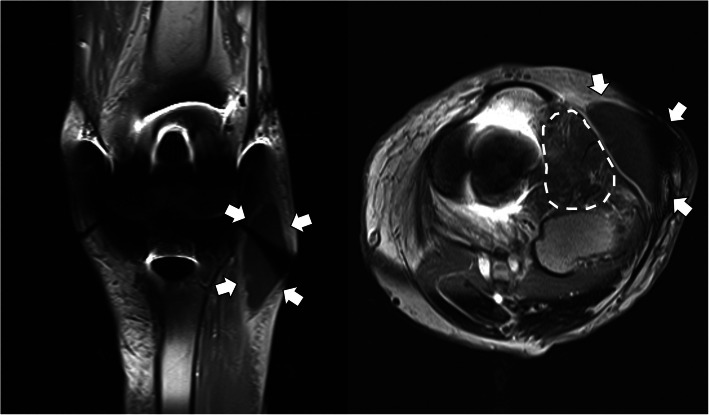
Contrast-enhanced T1-weighted magnetic resonance images of the left knee, showing destruction of the lateral tibial condyle filled with granulation tissue (dotted line) extending to adjacent subcutaneous abscess lesion (arrows) on the anterolateral aspect of the knee joint

At the time of admission, the patient’s vital signs were stable, with blood pressure of 130/70 mmHg, pulse rate of 76 beats/minute, and body temperature of 36.8 °C. On physical examination, there was an erythematous, swollen, warm, and tender lesion measuring 3 cm × 5 cm on the anterolateral aspect of the left knee. Laboratory tests revealed a white blood cell count of 5460 cells/mm^3^ with 50.7% neutrophils, erythrocyte sedimentation rate of 49 mm/h (reference range 0–20), and C-reactive protein of 15.73 mg/L (reference range 0–5). All other blood chemistry results were within the reference ranges. Radiography of the left knee at admission showed loosening of prosthesis owing to bone resorption of the proximal tibia and distal femur (Fig. 1). Synovial fluid analysis revealed the following: white blood cell count of 608 cells/mm^3^ (neutrophils 54%, lymphocytes 22%, macrophages 24%), red blood cell count of 2737 cells/mm^3^, glucose of 7 mg/dL, protein of 5.9 g/dL, and lactate dehydrogenase of 6547 IU/L. Magnetic resonance imaging demonstrated destruction of the lateral tibial condyle, filled with granulation tissue extending to adjacent diffuse cellulitis with a large abscess on the anterolateral aspect of the left knee (Fig. 2). Routine bacterial culture of the synovial fluid using the Vitek 2 microbial identification system (BioMerieux, France) was positive for *C. pelliculosa*. Results of other microbiological tests including acid-fast bacilli staining, mycobacterial culture, and real-time PCR assay to detect *Mycobacterium tuberculosis* and non-tuberculous mycobacteria were all negative.

The isolate was confirmed as *C. pelliculosa* using internal transcribed spacer 1 (5′-TCCGTAGGTGAACCTGCGG-3′) and 4 (5′-TCCTCCGCTTATTGATATG-3′) region PCR, followed by sequencing. Minimum inhibitory concentrations for antifungal agents were determined according to the Clinical and Laboratory Standards Institute guidelines by broth microdilution, and the results are as follows: 0.125 mg/L for amphotericin B, 0.06 mg/L for anidulafungin, 0.125 mg/L for caspofungin, 1 mg/L for fluconazole, 0.06 mg/L for micafungin, and 0.06 mg/L for voriconazole.

**Fig. 3:**
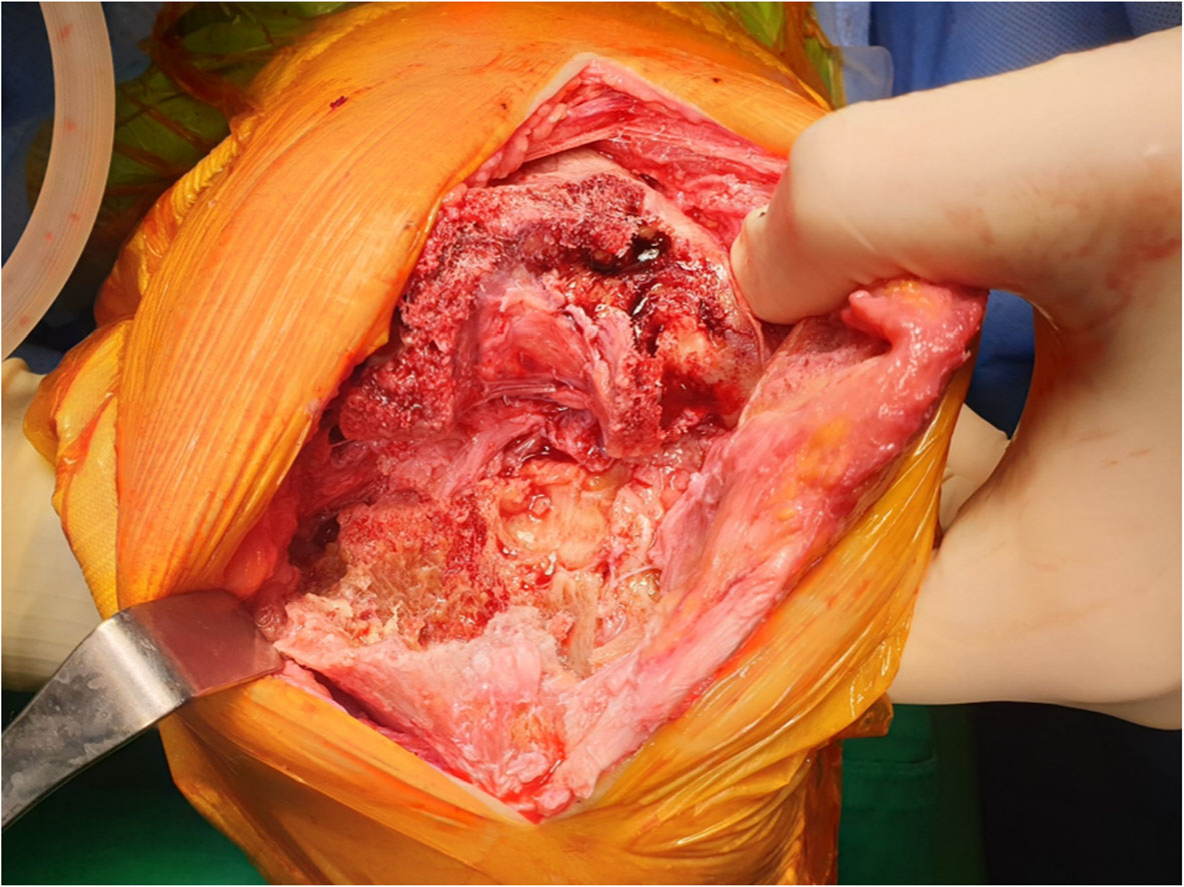
Operative findings of the left knee. Using midline skin incision and medial parapatellar approach to expose the left knee joint, femoral and tibial components of the prosthesis were removed, exposing infected granulation tissue and bone resorption mainly on the lateral tibial and femoral condyles

**Fig. 4:**
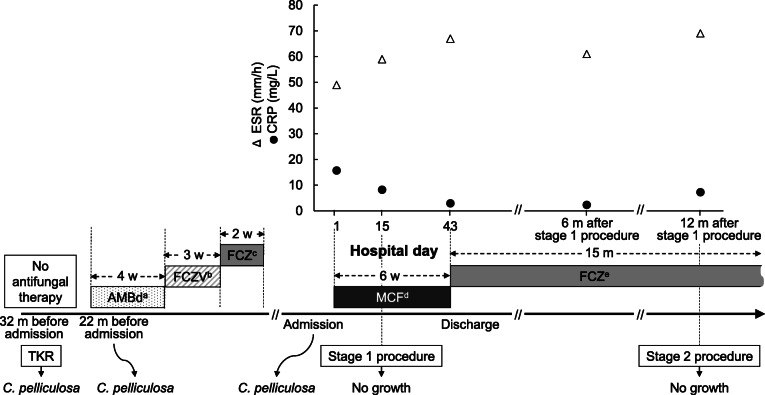
The patient’s medical history, clinical course, and treatment. ^a^ 0.5 mg/kg/day, ^b^ 6 mg/kg/day, ^c^ 4 mg/kg/day, ^d^ 100 mg/day, ^e^ 6 mg/kg/day. *TKA* total knee arthroplasty, *m* month(s), *w* week(s), *AMBd* amphotericin B deoxycholate, *FCZV* intravenous fluconazole, *FCZ* oral fluconazole, *MCF* micafungin

After 2 weeks of micafungin therapy (100 mg/day), the patient underwent surgery for prosthesis removal with debridement of soft tissue and bone, total synovectomy, and placement of an antibiotic-impregnated spacer. Operative findings showed infected granulation tissue in the whole joint space and bony fistula, extending to the anterolateral aspect of the lateral tibial component (Fig. 3). Histopathology showed acute and chronic inflammation with fibrinous exudation and necrotic debris. There was no isolated pathogen from intraoperative specimens. After the surgery, the patient was given micafungin for 4 weeks, followed by oral fluconazole (6 mg/kg/day). There were no signs of infection around the left knee, and the patient was discharged from the hospital. New prosthesis was implanted 12 months after the stage 1 procedure, and the patient is on taking oral fluconazole (6 mg/kg/day) and is scheduled to maintain for up to 3 months after the stage 2 procedure (Fig. 4). The prolonged antifungal therapy with oral fluconazole was well tolerated, and the patient was very compliant with the treatment.

## Discussion and conclusions

In this case, it is presumed that *C. pelliculosa* was inoculated at the time of intra-articular injection. However, *C. pelliculosa* isolated from the synovial fluid was initially disregarded as contamination in the patient who had complaints of chronic joint pain and swelling. After one year, *C. pelliculosa* was repeatedly identified from the same joint and antifungal treatment was initiated at a suboptimal dose, but the joint infection had extended to the adjacent bone, and eventually, prosthesis removal and prolonged use of antifungal agents were required.

With recent advances in medical technology, the incidence of *Candida* sp. osteoarticular infection has been steadily increasing over the last two decades [8–10]. *Candida* sp. osteoarticular infection is more often observed in immunocompromised hosts but may sometimes occur in normal hosts. Risk factors for *Candida* sp. osteoarticular infection include abdominal or orthopedic surgery, alcohol abuse, central venous catheters, diabetes mellitus, hemodialysis, hyperalimentation, immunosuppressive therapy, intravenous drug use, malignancy, prolonged use of broad-spectrum antimicrobials, and trauma or open wounds [1, 8, 10]. In most cases, *Candida* sp. osteoarticular infection results from hematogenous dissemination. It can also occur from direct inoculation during trauma, surgery, or intra-articular injections. In the former cases, symptoms associated with joint inflammation and fever may arise acutely. In contrast, patients with the latter cases, such as this case, often complain of an insidious onset of joint pain with minimal systemic manifestations. It is therefore not easy to diagnose *Candida* sp. osteoarticular infection early in patients who do not have a recent or current episode of candidemia at the time of presentation as well as a risk factor for *Candida* sp. osteoarticular infection. Patients with subtle symptoms and signs of joint infection without fever often experience delayed diagnosis. Delayed diagnosis of *Candida* sp. osteoarticular infection can lead to progressive joint destruction with irreversible loss of articular function.

There is no specific symptom or test for *Candida* sp. osteoarticular infection. The diagnosis can be established by culture of the synovial fluid. The most common species is *Candida albicans*, followed by *Candida tropicalis*, and *Candida parapsilosis*. *C. pelliculosa* (anamorph form), formerly known as *Pichia anomala* (teleomorph form), is a heterothallic, ascomycetous yeast. The recent taxonomic developments of yeasts have renamed *P. anomala* as *Wickerhamomyces anomalus* based on phylogenetic analysis on multigene sequences [11]. It is a rare, opportunistic pathogen with low virulence that exists widely in the environment. However, it has been recently reported as an emerging non-*Candida albicans* sp. associated with various forms of invasive infections [3–6]. In addition, there are several reports of nosocomial outbreaks of fungemia caused by *C. pelliculosa* in neonatal or pediatric intensive care units [12–14]. The major concern regarding treatment of infections caused by the rare yeast species is selecting optimal antifungal agents and dosages because there are currently no established breakpoints for these species. Although the data are limited, treatment with amphotericin B or fluconazole for *C. pelliculosa* infections has been reported to be successful [12–14]. In a more recent study of a nosocomial outbreak of fungemia caused by *C. pelliculosa*, the authors suggested echinocandin as an effective option for the treatment of *C. pelliculosa* infection [6].

For *Candida* sp. arthritis, fluconazole for 6 weeks or echinocandin (micafungin, caspofungin, or anidulafungin) for 2 weeks, followed by fluconazole for at least 4 weeks is recommended [15]. However, the duration of antifungal therapy should be extended to 6–12 months for *Candida* sp. osteomyelitis [15]. For *Candida* sp. arthritis of native joints, adequate drainage is recommended [15]. However, in one study, only 36% of patients underwent adjunctive surgical intervention including drainage, irrigation, debridement, and amputation, and 62% of patients were treated with medical therapy alone [10]. In most cases with *Candida sp.* osteomyelitis, both surgical intervention and prolonged antifungal therapy are warranted to eradicate the infection [16].

*Candida* sp. prosthetic joint infection is more difficult to treat. The formation of *Candida* biofilms on both human tissues and prosthetic materials has been studied extensively and is thought to contribute to treatment failure [17–19]. For the treatment of *Candida* sp. prosthetic joint infection, a two-stage arthroplasty exchange is the preferred surgical approach for eradicating infection and preserving joint function [15, 20]. Two-stage arthroplasty exchange is composed of two surgeries: removal of all prosthetic materials (stage 1), and re-implantation of a new prosthesis usually 3 to 6 months later (stage 2). Antifungal therapy should be continued for at least 12 weeks after stage 1 surgery and for at least 6 weeks after stage 2 surgery. The interval between the two surgeries is not yet established.

A rare *Candida* sp. could be a causative organism of osteoarticular infection, especially in patients with a prior history of intra-articular injections. Even a single colony of *Candida* on synovial fluid should be considered as pathogenic, and the patient should receive the optimized antifungal therapy by selecting the appropriate drug, dosage, and duration. A high index of suspicion is required to establish the correct diagnosis and successful treatment to prevent irreversible joint destruction and preserve articular function.

## Data Availability

The data used during the current study are available from the corresponding author on reasonable request.
